# Outcome of patients with traumatic cranial nerve palsy admitted to a university hospital in Nepal

**DOI:** 10.1186/s41016-024-00361-8

**Published:** 2024-04-01

**Authors:** Khusbu Kumari, Naveen Gautam, Monika Parajuli, Shreejana Singh, Amit Pradhananga, Gopal Sedai, Sushil Shilpakar, Mohan Raj Sharma

**Affiliations:** 1 Maharajgunj Medical Campus, Institute of Medicine, Tribhuvan University, Maharajgunj, Kathmandu, Nepal; 2 Department of Neurosurgery, Tribhuvan University Teaching Hospital, Maharajgunj Kathmandu, Nepal; 3 Department of Research, Institute of Medicine, Maharajgunj Kathmandu, Nepal

**Keywords:** Cranial nerve palsy, Nepal, Outcome, Traumatic brain injury

## Abstract

**Background:**

Cranial nerve palsy (CNP) is a common complication of traumatic brain injury (TBI). Despite a high incidence of TBI in Nepal (382 per 100,000), literature on the specific management and outcome of CNP is lacking. This study aimed to examine the outcomes of TBI patients involving single versus multiple CNP.

**Methods:**

A retrospective chart review of 170 consecutive TBI patients admitted to the tertiary neurosurgical center in Nepal between April 2020 and April 2022 was conducted. Demographic, clinical, and etiological characteristics; imaging findings; and management strategies were recorded, compared, and analyzed using descriptive statistics. The Glasgow Outcome Scale Extended (GOSE) was used to measure the outcomes in two groups of patients (single and multiple CNP) at 3 months.

**Results:**

Out of 250 eligible patients, 80 were excluded and CNP was noted in 29 (17.1%) of the remaining 170. The median age was 34.9 years, and falls (60.6%) were the most common cause of trauma. TBI severity was categorized based on GCS: mild (82.4%), moderate (15.9%), and severe (1.8%). Cranial nerve involvement was seen in 29 (17.05%) patients: single cranial nerve involvement in 26 (89.65%) and multiple nerve involvement in 3 (10.34%). The most common isolated cranial nerve involved was the oculomotor nerve (37.9%). CT findings revealed a maximum of skull fractures with no significant association between CNP and CT findings.

**Conclusions:**

CNP is a common consequence of TBI with the most common etiology being falls followed by RTA. Single CNP was more common than multiple CNP with no significant difference in the outcome in the 3-month GOSE score. Further research is needed to determine the burden of traumatic CNP and establish specific management guidelines for different types of CNP.

## Background

Traumatic brain injury (TBI) is a multifaceted condition that encompasses a wide range of injuries resulting from external physical trauma to the brain. It is one of the leading causes of death and disability in Nepal, with an estimated incidence of 382 per 100,000 individuals, which is higher than the global average of 351 per 100,000 [1]. It can manifest in various forms, ranging from mild, such as a concussion, to severe, potentially culminating in a state of coma or death [2]. The etiology of TBI is varied, with common causes including road traffic accidents (RTAs), falls, and physical assaults. Additionally, TBI is associated with cranial nerve palsy (CNP) in a significant proportion of cases, characterized by a diminution or total cessation of the functions of one or more cranial nerves.

The demographic and clinical characteristics, as well as the outcomes of patients suffering from CNP in the context of TBI, have been well-documented in the extant literature [3, 4]. However, despite the high prevalence of TBI in Nepal, there exists a dearth of literature on the specific incidence and prognosis of CNP associated with TBI. To address this gap, this study aimed to conduct a retrospective review of TBI patients admitted to Tribhuvan University Teaching Hospital (TUTH), a tertiary care center in Nepal.

## Methods

A retrospective chart review of 170 consecutive patients who sustained head trauma between April 12, 2020, and April 12, 2022, was conducted. Approval from the Institutional Review Committee (IRC) [Reference number: 488(6-11)e2078/079] was obtained before data collection. Patients who were lost to follow-up left against medical advice or had missing data were excluded from the study.

Patients were managed according to standard departmental protocols and Brain Trauma Foundation guidelines (fourth edition) [5] for moderate to severe traumatic brain injury. This included optimization of blood pressure and oxygenation, close neurological monitoring with prompt repeat head CT imaging for any decline ≥ 2 points on the Glasgow Coma Scale (GCS). The study population was stratified into those with and without CNP. Management did not differ between these groups; no surgical interventions were undertaken specifically to address cranial nerve palsies. The adult version of the Glasgow Outcome Scale Extended (GOSE) [6] was utilized to assess the outcomes for patients over 16 years old, while the pediatric version was used for those under 16 years old.

The analysis was conducted using Microsoft Excel 365. The study employed descriptive statistics to summarize the demographic, clinical, and imaging factors as means, medians, and proportions.

## Results

Out of 250 patients eligible to participate during the study period, 80 patients were excluded either due to missing data or loss to follow-up due to the COVID-19 pandemic. We elected not to recruit patients retrospectively from the pre-pandemic period to avoid introducing confounds from potentially different patient populations. Out of the remaining 170, CNP was noted in 29 (17.1%) patients.

### Demographics

Of 170 patients, 124 (72.9%) were males, and 46 (27.05%) were females. The median age was 34.9 years ranging from 8 months to 88 years.

### Etiology

Fall [103 (60.6%)] was the most common cause of trauma, followed by road traffic accidents (RTA) [43 (25.3%)], physical assault [14 (8.2%)], and other causes [9 (5.3%)].

### Clinical presentations

Details of the patient’s demographic characteristics, mechanism of injury, GCS at the time of admission, clinical diagnosis, CT findings, and CNP and GOSE at a 3-month follow-up can be seen in Table 1. TBI severity was categorized based on the Glasgow Coma Scale (GCS) as mild (GCS 13–15), moderate [7–9], or severe 3–6, 10, 11]. One hundred forty (82.4%) patients had mild TBI, 27 (15.9%) had moderate, and 3 (1.8%) had severe TBI. A non-reactive pupil was seen in 14 (8.23%) patients. Table 2 shows the neurological and non-neurological symptoms of TBI associated with CNP.

**Table 1:** Demographic, clinical characteristics, management, and outcome of patients with CNP

S.N	Age	Sex	Mechanism of injury (MOI)	GCS	Clinical diagnosis	CT scan findings	CNP	Management strategy	Outcome at 3 months (GOSE scale)^a^
1	34	F	RTA	12	Moderate	Grossly normal	III	Non-operative	1
2	45	F	RTA	15	Mild	SDH, contusion	III	Operative	1
3	50	F	Others	15	Mild	EDH	III	Non-operative	7
4	11	F	Fall	15	Mild	Contusion	III	Non-operative	0
5	21	M	Physical assault	14	Mild	Contusion, skull fracture	VII	Non-operative	1
6	26	M	Physical assault	15	Mild	Skull fracture	III	Non-operative	8
7	40	M	Fall	15	Mild	EDH	III	Operative	8
8	23	M	Physical assault	15	Mild	Skull fracture, contusion	VII	Non-operative	7
9	33	F	RTA	15	Mild	EDH, SAH	III	Operative	7
10	24	M	Fall	15	Mild	EDH, skull fracture	I	Non-operative	8
11	85	M	Fall	12	Moderate	SDH	VII	Operative	8
12	30	M	Others	15	Mild	EDH	VII	Operative	7
13	42	F	Fall	9	Moderate	SDH	III	Operative	8
14	23	F	Fall	7	Severe	SDH	II, III, VII	Operative	6
15	40	M	Fall	15	Mild	SDH	II	Operative	7
16	40	M	Physical assault	15	Mild	Miscellaneous (pneumocephalus, ICH hematoma, DAI)	II	Non-operative	7
17	32	M	RTA	8	Severe	Skull fracture, EDH	II, III	Operative	7
18	45	M	Fall	15	Mild	Contusion, SDH	III	Operative	7
19	29	M	RTA	15	Mild	Skull fracture	III	Non-operative	8
20	27	M	RTA	13	Mild	Skull fracture	II	Operative	9
21	45	M	Physical assault	15	Mild	ICH	VII	Non-operative	8
22	24	M	Physical assault	15	Mild	Skull fracture	II	Operative	7
23	46	M	Fall	9	Moderate	SDH	VII	Operative	8
24	77	M	Fall	15	Mild	EDH, skull fracture	II	Operative	7
25	83	M	Fall	11	Moderate	SDH	II	Non-operative	6
26	24	M	Physical assault	15	Mild	Skull fracture	II	Operative	8
27	56	M	Fall	12	Moderate	Others (pneumocephalus, ICH hematoma, DAI)	VII	Non-operative	5
28	59	F	Fall	9	Moderate	EDH	III	Operative	2
29	2	M	Fall	15	Mild	Contusion, SDH, skull fracture	II, III	Non-operative	9

^a^GOSE scale referring to 1 = death, 2 = persistent vegetative state, 3 = lower severe disability, 4 = upper severe disability, 5 = lower moderate disability, 6 = upper moderate disability, 7 = lower good recovery, 8 = upper good recovery, and 9 = missing

**Table 2:** Symptomatology in CNP

**Neurological symptoms**	
Ocular symptoms (RAPD, blurred vision, lagophthalmos, decreased visual acuity)	3
Decreased power	1
Aphasia	2
Seizure	3
Miscellaneous (vertigo, ataxia, bladder symptoms)	20
**Systemic symptoms**	
ENT bleed	3
Chest and abdomen and extremity injury	2
Generalized unspecified injuries (including contusions, lacerations, fever)	24

### Cranial nerve palsies (CNP): single vs multiple

Cranial nerve involvement was seen in 29 patients, out of which single cranial nerve involvement was noted in 26 patients (89.65%) patients while 3 (10.34%) patients had multiple nerve involvement. As shown in Table 1, the most common isolated cranial nerve involved was oculomotor nerve 11 (37.9%) followed by the same proportion of facial 7 (24.1%) and optic nerve 7 (24.1%). Among the multiple CNP, optic and oculomotor nerves were simultaneously involved in 2 patients, and 1 patient had concurrent involvement of optic, oculomotor, and facial nerves.

### CT scan findings

CT findings revealed a maximum of skull fractures 11 followed by EDH with 10 cases. There was no significant association between the type of CNP and the CT findings.

### Management strategy

Surgical/operative intervention (evacuation of hematoma) was performed in 16 patients (55.17%) with CNP, while 13 (44.8%) patients underwent non-operative management through conservative measures (e.g., raised ICP management, use of steroids). The mean length of hospital stay was 13.86 days, and the mean duration of ICU stay was 4.41 days. Out of 26 single CNP, 12 (46.15%) were managed non-operatively, while the rest 14 (53.8%) underwent operative intervention. The most commonly involved single CNP viz. oculomotor nerve palsy was mostly managed operatively, 6 (54.54%), with the remaining 5 (45.45%) managed non-operatively. Two cases of multiple CNP (optic-oculomotor palsy and optic-oculomotor-facial palsy) were managed operatively with 1 case (optic-oculomotor palsy) managed non-operatively.

### Outcome of isolated CNP vs multiple CNP

**Fig. 1:**
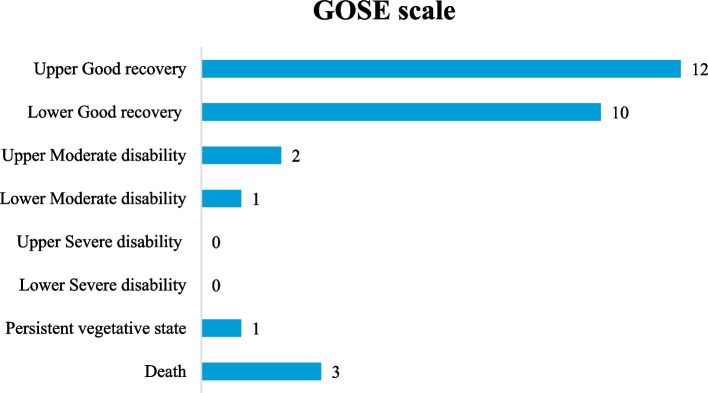
GOSE scale findings among CNP patients

The Glasgow Outcome Scale Extended (GOSE) was utilized to assess the 3-month post-treatment outcomes. Fig. 1 shows the outcome of both single and multiple CNP in the form of a chart. As per the figure, a single CNP had 3 deaths (GOSE score 1), 2 with oculomotor nerve involvement, and 1 with facial nerve involvement. It also had 11 upper good recoveries (GOSE score 8). Multiple CNP, however, had GOSE scores of 6 (upper moderate recovery), 7 (lower good recovery), and 8 (upper good recovery) in one patient each.

## Discussion

CNP is one of the common consequences of TBI. The literature [10, 11] conducted across multiple countries has assessed its prevalence which ranges from 1 to 23%; however, it is not extensively studied in the context of a lower-middle country like Nepal. Single CNP is more common than multiple CNP [7]. Our study is the first of its kind that assesses the clinical characteristics, etiology, clinical presentations, CT scan findings, management strategy, and the pertinent difference in outcomes between single and multiple CNP in the largest neurosurgical tertiary care center in Nepal involving 170 patients with TBI.

### Demographics

Almost one-third of patients sustaining TBI recorded in our center were males. The median age of TBI patients was middle-aged working adults concordant with other literature [8] with a very wide age range. Epidemiological studies indicate that men are 2.22 times more likely than women to sustain a TBI [8, 9]. The exact reason for this gender propensity is not yet fully understood [12]. However, some studies suggest that hormonal and biological variations [9] such as increased testosterone in men leading to aggressive and higher risk-taking behavior may play a role [13].

### Etiology

Our study found that fall was the most common cause of TBI, accounting for over half of the cases, followed by road traffic accidents. This distribution is similar to the findings from other studies globally [14, 15]. Falls are more likely to occur in elderly populations [16], which may have contributed to the predominance observed in our cohort. Analyses of injury severity patterns suggest RTAs are often more deadly than falls [17]. Thus, our hospital-based study could underestimate traffic-related cranial nerve injuries if less survivable cases were missed. However, road safety metrics have improved after Nepal adopted strict drunk driving policies in 2011 [17], leading to fewer alcohol-related severe and fatal injuries nationwide based on transport authority records. Concurrently, increased urban congestion may be precipitating more frequent mild road traffic injuries that do not present for care.

A subgroup analysis evaluated the association between injury mechanism and risk of TBI with or without concomitant CNP. Among 103 fall-related cases, 14 patients (13.6%) had CNP, while of 43 patients sustaining RTA, 6 (13.9%) developed CNP. The odds ratio for CNP was 0.969 when comparing fall and RTA etiologies, indicating no statistically significant difference for secondary nerve injury across the predominant mechanisms of injuries.

### Clinical presentations

Upon categorizing the severity of TBI using the GCS scale [18], more than 80% of the hospitalized patients had mild TBI (GCS 14–15) with only a very few proportions of severe TBI (GCS < 8) which is similar to other literature [19]. TBI associated with CNP was seen in about one-fifth of patients out of which single cranial nerve involvement was the most common accounting for almost 90% of the cases. The literature on CNP following TBI gives a wide range (1 to 23%) [10, 11] of the estimated prevalence across the globe. However so, most of those studies were carried out in higher-income countries, and similar literature in the context of lower-middle-income countries is severely lacking, thus warranting further needs of research in this area.

The most common isolated cranial nerve involved was the oculomotor nerve followed by the facial nerve. Although isolated oculomotor nerve involvement in TBI is relatively rare in the case of mild TBI [20], severe TBI can occasionally result in it [20]. The hospitalized patient in our center had endured a higher proportion of mild TBI, but the isolated oculomotor nerve was seen to be the most commonly involved cranial nerve, which contrasts with other literature [20, 21]. The optic-oculomotor combination was the most commonly involved multiple cranial nerves in our study. A 2005 study, analyzed out of 979 cases [22], revealed the combination of oculomotor and abducens, trochlear and abducens, or trochlear and facial to be the most common variants among multiple CNP.

Ocular symptoms (RAPD, blurred vision, lagophthalmos, decreased visual acuity) were the most common neurological symptoms seen in CNP patients.

### CT scan findings

The CT findings in our study revealed about 1/3rd of cases of skull fractures with one fracture of the skull base in the CNP patients. But the exact distinction between the type of CNP and the corresponding CT finding could not be established from our study. CT scans have been useful for assessing the fracture extension and intraosseous cranial nerve segment [23]. We could not identify any literature that compares the type of CNP with the CT findings and the correlation between the two. This necessitates more research in this field to establish the exact data regarding the same.

### Management strategy

More than 50% of the hospitalized patients in our center underwent surgical exploration (i.e., hematoma evacuation) for CNP rather than non-surgical intervention. Single CNP most commonly underwent hematoma evacuation. One study on the efficacy of surgical decompression together with evacuation of hematoma of post-traumatic facial nerve palsy showed a robust outcome for the patient after the procedure [24]. We could not find adequate literature that establishes a clear guideline for the definitive management of CNP. However, most of the literature suggests that prompt recognition, rapid resuscitation, and definitive operative management of the cases are of paramount importance to prevent secondary brain injuries [21]. At our center, most cranial nerve injuries were managed conservatively with favorable functional outcomes. Optic nerve injuries associated with severe brain injury were managed with steroids. However, there is an ongoing debate about whether or not steroid alters any management-related outcome [25, 26]. Further research is needed to establish the preferential mode of management.

In our center, oculomotor nerve injury, the most common CNP, was managed conservatively. About 3/4 of patients improved within 3 months, with only a few developing residual palsy not affecting their daily lives. Currently, a controversy prevails regarding the non-surgical vs surgical mode of management of traumatic oculomotor nerve [27, 28]. This too calls for more well-designed research studies for the establishment of clear management guidelines.

Our study involved a conservative approach with steroids for the management of facial nerve injuries. Although a complete recovery was noted in about 3/4th of the patients in our center within 3 months, the lack of establishment of a clear consensus in the management of facial nerve injuries via surgical or non-surgical approach remains a topic of further discussion [29].

### Outcome of isolated CNP vs multiple CNP

GOSE scale [6] was used for comparison of outcomes between the two groups with single vs multiple CNP. The study shows that a GOSE score of 1 (death) was all seen with single CNP patients with upper good recovery seen in about 1/3 of cases. This does not clearly outline the outcome between the GOSE and the type of CNP involved. No papers establishing a distinct difference in outcome between single and multiple CNP could be identified, hence warranting more focus in this area in future studies as well.

The limitations of the study were small sample sizes and reliance on self-reported data. This indicates a need for larger, multi-centric studies with a control group and longitudinal follow-up to establish effective management protocols. Randomized controlled trials establishing the efficacy of current management protocols specific to Nepal are necessary. Overall, this study serves as a guide for future research and highlights the need for further investigation into traumatic CNP in the context of a lower-middle-income country like Nepal.

### Limitations and future directions

Owing to the study being conducted during the COVID-19 pandemic, the final sample size was smaller than originally planned due to missing data and loss to follow-up. We could not retrospectively enroll patients from the pre-pandemic period, which may have represented different patient demographics. However, we believe examining 29 patients with TBI and cranial nerve involvement out of 170 TBI patients provides perspective on the management and outcomes of this population in Nepal. The authors acknowledge the study’s limitations including the modest sample size and challenges posed by the COVID-19 pandemic resulting in intermittent government-enforced lockdowns and patients’ hesitancy to seek hospital visits unless in urgent situations, potentially impacting the completeness and accuracy of long-term data. Moreover, the 3-month patient follow-up was constrained by feasibility and the time-limited nature of the principal author-led analysis. Undoubtedly, the more extended follow-up would better delineate the functional recovery trajectories and outcomes of both single and multiple traumatic cranial neuropathies.

This paper intends to serve as a foundational basis for future expanded multi-center research aimed at accumulating larger sample sizes, comprehending national data trends, and shaping context-specific management guidelines. A more comprehensive prognostic model integrating additional predictors such as frailty status and longer follow-up periods would further enhance the paper’s strength.

## Conclusions

This study demonstrates that traumatic CNP is a significant complication following TBI, with the majority of patients with single nerve involvement. There was no significant difference in the outcomes between single and multiple CNP concerning clinical characteristics, etiology, CT findings, management protocols, and GOSE. Further research is needed to outline the exact burden of traumatic CNP and establish a clearer definite management guideline regarding the management of various types of CNP in our context.

## Data Availability

The data used in this study was collected from the Tribhuvan University Teaching Hospital (TUTH) medical record section. The raw data can be made available upon request to the corresponding author. Any further material necessary for the replication of this study can also be made available upon request.

## Abbreviations

CNP: Cranial nerve palsy
CT: Computed tomography
EDH: Epidural hematoma
GCS: Glasgow coma scale
GOSE: Glasgow outcome scale extended
ICH: Intracerebral hemorrhage
ICU: Intensive care unit
INGOs: International non-governmental organizations
IRC: Institutional Review Committee
NGOs: Non-governmental organizations
RTAs: Road traffic accidents
SAH: Subarachnoid hemorrhage
SDH: Subdural hematoma
TBI: Traumatic brain injury
TUTH: Tribhuvan University Teaching Hospital
